# Poverty dynamics and health in late childhood in the UK: evidence from the Millennium Cohort Study

**DOI:** 10.1136/archdischild-2018-316702

**Published:** 2019-06-11

**Authors:** Eric T C Lai, Sophie Wickham, Catherine Law, Margaret Whitehead, Benjamin Barr, David Taylor-Robinson

**Affiliations:** 1 Department of Public Health and Policy, University of Liverpool, Liverpool, UK; 2 UCL Great Ormond Street Institute of Child Health, London, UK

**Keywords:** child poverty, trajectories, mental health, physical health, longitudinal study

## Abstract

**Objective:**

To assess the prevalence of different trajectories of exposure to child poverty and their association with three indicators of adolescent physical and mental health in UK children.

**Methods:**

We analysed data on 10 652 children from a large, prospective, nationally representative sample in the UK Millennium Cohort Study. The outcomes were mental health, measured by the Strengths and Difficulties Questionnaire (SDQ), physical health, measured by obesity and any longstanding illness, at age 14. The exposure was relative poverty (<60% of median of equivalised household income), measured at 9 months, 3, 5, 7, 11 and 14 years. Poverty trajectories were characterised using latent class analysis. ORs and 95% CIs were estimated using multivariable logistic regression, adjusted for maternal education and ethnicity.

**Results:**

Four poverty trajectories were identified: never in poverty (62.4%), poverty in early childhood (13.4%), poverty in late childhood (5.0%) and persistent poverty (19.4%). Compared with children who never experienced poverty, those in persistent poverty were at increased risk of mental health problems (SDQ score≥17 (adjusted OR (aOR): 3.17; 95% CI: 2.40 to 4.19)), obesity (aOR: 1.57; 95% CI: 1.20 to 2.04) and longstanding illness (aOR: 1.98; 95% CI: 1.55 to 2.52). Poverty in early childhood was related to higher risk of obesity than that in late childhood, while the opposite is observed for mental health problems and longstanding illness.

**Conclusions:**

Persistent poverty affects one in five children in the UK. Any exposure to poverty was associated with worse physical and mental health outcomes. Policies that reduce child poverty and its consequences are likely to improve health in adolescence.

**What is already known on this subject?**

- Child poverty is increasing in the UK.
- Strong evidence suggests that the effects of child poverty on adverse child health outcomes are causal.

**What this study adds?**

- Our analysis of a representative UK children’s cohort following up from 2000 to 2016 found that one in five children were in persistent poverty up to age 14 years.
- Any exposure to poverty was associated with worse physical and mental health in early adolescence.
- Ending child poverty should become a policy priority in order for UK children to achieve their full potential.

## Introduction

Child poverty is rising in the UK; 30% of children were living in poverty (defined as having a household income after housing costs of 60% below the median income) in 2016–17 (a total of 4.1 million children), an increase from 27% in 2010–11.^1^ The proportion of children living in poverty is projected to continue to rise over the next 5 years.^2^ Persistent poverty has been shown to be related to poorer cognitive, social and behavioural development of the children,^3 4^ as well as poorer self-rated well-being for both mothers and children.^5^ There is strong evidence to suggest the association of child poverty with worse health outcomes is causal.^6 7^ A recent systematic review by Cooper and Stewart^6^ demonstrated causal links between income poverty and school achievement, cognitive development, social and behavioural development and health in children. Importantly, these adverse health outcomes track into adulthood.^8^ Furthermore, poor adolescent health is associated with worse educational outcomes, employment status and socioeconomic position in adulthood.^9^

What remains less clear is whether specific patterns of exposure to poverty have different effects on particular aspects of child health. A small study in Quebec in Canada showed that, compared with families who never experienced poverty, those who experienced intermittent poverty was associated with a higher risk of asthma-like wheezing, poorer overall child health^10^ and higher chance of childhood overweight or obesity.^11^ Few studies, however, have assessed the prevalence and impact of specific poverty patterns that extend beyond early childhood. Here, we assess the prevalence of the differing patterns of poverty experienced by children in the UK and their association with three indicators of health in adolescence using data from a large cohort in the UK.

## Methods

### Study population

We used data from the Millennium Cohort Study (MCS), a large nationally representative cohort sample of children born in the UK between September 2000 and January 2002 who have been followed up through six survey waves, when the children were 9 months, 3, 5, 7, 11 and 14 years of age.^12 13^ The study oversampled children living in disadvantaged areas and in those with high proportions of ethnic minority groups by means of a stratified cluster sampling. Further information on the MCS is detailed elsewhere.^13^ Following our previous study,^14^ we included only singletons and those whose latest interview was conducted with the natural mother to ensure consistent reporting of variables.

### Exposure

The main exposure is relative income poverty at different times of follow-up. We defined poverty here as <60% of median household income where self-reported income (by parents in each wave of follow-up) is equivalised according to the Organisation for Economic Co-operation and Development household equivalence scale.^15^ The MCS survey team used multivariable interval regression to impute missing income data in all waves.^16 17^

### Outcomes

The main outcomes of interest were socioemotional behavioural problems, obesity and any longstanding illness at age 14 years. Children’s socioemotional behavioural problems were measured by Strengths and Difficulties Questionnaire (SDQ) on the basis of maternal report. The SDQ is a 25-item measure that asks parents to rate their child’s behaviour over the previous 6 months using five subscales, each with five items: peer problems, conduct disorders, hyperactivity, emotional problems and prosocial behaviour. We used the total difficulties score (which excludes the prosocial behaviour items) using validated cut-offs widely used in previous studies,^14 18^ that is, 0–16 indicates normal to borderline behaviour and 17–40 indicates socioemotional behavioural problems.^19^ The SDQ has good internal consistency (Cronbach’s α=0.77) in the study sample.^14^

The children’s anthropometrics were measured without shoes and with light clothing by trained interviewers. Standing height was measured to the nearest millimetre using a Leicester height measure stadiometer and a Frankfurt plane to assist measurements. Weight was measured using a Tanita BF-522W scale. Body mass index was calculated in kg/m^2^,^12^ and obesity categorised using the Internation Obesity Task Force (IOTF) cut-offs.^20^ Longstanding illness was measured by asking the mother in the interview ‘does (child) have any physical or mental health conditions or illnesses lasting or expected to last 12 months or more?’ We removed longstanding illnesses related to mental health in order to avoid double counting with SDQ scores.

### Covariates

Confounders were chosen based on common causes of both exposure (relative income poverty) and outcomes (child health outcomes) or potentially on the confounding pathway (online supplementary figure 1).^21^ We have adjusted for maternal education (highest academic and vocational qualifications achieved by mothers) and maternal ethnicity (‘white’, ‘mixed’, ‘Indian’, ‘Pakistani and Bangladeshi’, ‘black or Black British’ or ‘other ethnic group’ using the six-category census classification) as covariates, reported when the child was aged 9 months^22^ and lone parenthood at 9 months. Maternal education was reportedly related to both family income and children’s socioemotional behavioural problems in previous MCS studies.^23^ It has been shown in a previous MCS report that children from all ethnic minority groups have a higher risk of being persistently poor throughout childhood.^5^ Families with lone parents are more likely to be in persistent poverty than families with two parents,^5^ and children as a result would be more prone to socioemotional behavioural problems.^24^

10.1136/archdischild-2018-316702.supp1 Supplementary file 1

### Statistical analysis

We used two approaches to characterise patterns of exposure to poverty up to age 14 years: latent class analysis and a dose-response analysis. First, we characterised specific poverty trajectories, across all six time points of follow-up using latent class analysis with the *poLCA* package in R,^25 26^ which takes advantage of the iterative nature of the expectation-maximisation algorithm to estimate latent classes even when some observations of the manifest variables are missing,^25^ in order to assess if health outcomes are more sensitive to exposure to poverty at certain stage of development (sensitivity period hypothesis).^27^ We considered at least four trajectories to take full advantage of the large sample size of our cohort and to ensure sufficient granularity. Latent classes were generated using the binary variables indicating poverty from each wave. The model with four trajectories fits the data the best as informed by the lowest Bayesian information criterion value. We took a cut-off of probability of 0.5 to consider whether families in a particular trajectory at a given time are in poverty. Four poverty trajectories were characterised: never in poverty (reference group), poverty particularly in early childhood (age 9 months to 7 years), poverty particularly in late childhood/adolescence (age 11–14 years) and persistent poverty (highly likely to be in poverty in each wave (p>0.8)) (online supplementary figure 2).

Pearson’s Χ^2^ test was used to compare the baseline characteristics of the cohort participants across poverty trajectories. We assessed the associations between predicted poverty trajectories and socioemotional behaviour problems, obesity and longstanding illnesses using multivariable logistic regression, using ORs and 95% CIs. We built two models to observe the effect of confounders: model 1 is the crude model between poverty trajectories and each outcome and model 2 replicates model 1 additionally adjusted for maternal education and ethnicity. Both models included longitudinal weights, accounting for attrition, sampling design and the differential selection of particular types of ward in order to be representative of the national population of families with a child of this age. Collinearity of models were checked with variance inflation factors, with that exceed 5 indicating problematic collinearity.^28^

Whether poverty trajectories had different associations with child health and mental health outcomes by sex was assessed from the significance of interaction terms, since girls are more likely to report poorer mental health than boys,^29^ and weight gain and fat deposition start at different ages among boys and girls.^20^

We also assessed whether exposure to poverty displayed any dose-response relationship, that is, the cumulative hypothesis.^30–33^ We further coded the exposure to poverty as a cumulative score, that is, 0 means never identified as poverty and 6 means identified as poverty at all waves of follow-up. We then assessed the association of cumulative exposure to poverty and health outcomes at age 14. We have done several sensitivity analyses. First, we conducted a complete case analysis using only those with complete observation of exposure, outcome and covariates. Second, to account for (online supplementary table 1), we repeated the analysis with multiple imputed datasets.^34^ Third, to check the robustness of our poverty measure, we also repeated the analysis using subjective poverty, which was defined as whether the main respondent (natural mothers in this case) felt that they were just about getting by financially or worse in each wave of follow-up (subjective poverty).^35^ Analysis was undertaken using R (V.3.4.3).

### Ethics statement

Parents provided written informed consent for all components of MCS. At the age 14 follow-up, children also provided informed consent.

## Results

### Poverty trajectories

A total of 15 415 families took part in the MCS when the children were aged 14 (wave 6), 11 726 families responded to the questionnaire. Including only singletons and those whose interview was conducted with natural mothers, and where poverty was recorded in at least one wave yielded a sample of 10 652 children (online supplementary figure 3). Prevalence of poverty in each wave of follow-up was given in online supplementary table 2. Table 1 shows the baseline characteristics of the included children stratified by poverty trajectories; 19.2% of children were in persistent poverty across all waves of follow-up, whereas 62.4% of children were never in poverty across all waves. The remaining 13.4% and 5.0% of them were in poverty particularly in early childhood and in late childhood/adolescence, respectively. All of the interested covariates differed among different trajectories. More girls than boys were in poverty in early childhood, whereas it was the opposite for poverty in late childhood. Mothers of children in poverty in late childhood and persistent poverty had poorer education than the other two trajectories and were more likely to be of non-white ethnicity.

**Table 1:** Baseline characteristics of the cohort participants in the UK Millennium Cohort Study in wave 6 (age 14)

Characteristics	Predicted poverty trajectories				
	Never in poverty* (n=6652)	Poverty in early childhood (n=1424)	Poverty in late childhood (n=530)	Persistent poverty (n=2046)	P value
Child’s sex					0.04
Boy	3276 (50.4%)	642 (47.0%)	273 (53.6%)	952 (49.5%)	
Girl	3221 (49.6%)	723 (53.0%)	236 (46.4%)	971 (50.5%)	
Maternal education					<0.001
Higher degree	397 (6.1%)	20 (1.5%)	1 (0.2%)	0 (0%)	
First degree	1518 (23.4%)	68 (5.0%)	8 (1.6%)	18 (0.9%)	
Diploma	849 (13.1%)	76 (5.6%)	30 (5.9%)	27 (1.4%)	
A-levels	814 (12.5%)	132 (9.7%)	33 (6.5%)	56 (2.9%)	
GCSE A–C	2119 (32.6%)	551 (40.4%)	190 (37.4%)	523 (27.4%)	
GCSE D–G	414 (6.4%)	224 (16.4%)	84 (16.5%)	289 (15.1%)	
None	382 (5.9%)	293 (21.5%)	162 (31.9%)	996 (52.2%)	
Maternal ethnicity					<0.001
White	6017 (92.8%)	1146 (84.1%)	387 (76.2%)	1152 (60.1%)	
Mixed	34 (0.5%)	18 (1.3%)	8 (1.6%)	32 (1.7%)	
Indian	158 (2.4%)	55 (4.0%)	7 (1.4%)	43 (2.2%)	
Pakistani and Bangladeshi	60 (0.9%)	48 (3.5%)	67 (13.2%)	541 (28.2%)	
Black or Black British	120 (1.9%)	61 (4.5%)	28 (5.5%)	117 (6.1%)	
Other ethnic groups	95 (1.5%)	35 (2.6%)	11 (2.2%)	33 (1.7%)	
Outcomes					
Socioemotional behavioural problems					<0.001
SDQ score<17	6102 (94.1%)	1220 (87.9%)	416 (80.6%)	1589 (81.8%)	
SDQ score≥17	381 (5.9%)	168 (12.1%)	100 (19.4%)	353 (18.2%)	
Obesity					<0.001
Not obese	5997 (94.7%)	1200 (90.2%)	432 (89.1%)	1652 (88.3%)	
Obese	335 (5.3%)	130 (9.8%)	53 (10.9%)	219 (11.7%)	
Longstanding illness					<0.001
No	5684 (87.4%)	1162 (84.9%)	405 (79.1%)	1644 (84.0%)	
Yes	821 (12.6%)	207 (15.1%)	107 (20.9%)	312 (16.0%)	

*‘Never in poverty’ is a description of the overall class and that some of these children might have been in poverty at some waves, but with very low probability (see online supplementary figure 2).

SDQ, Strengths and Difficulties Questionnaire.

### Poverty trajectories and health in childhood: assessing sensitive periods

**Figure 1:**
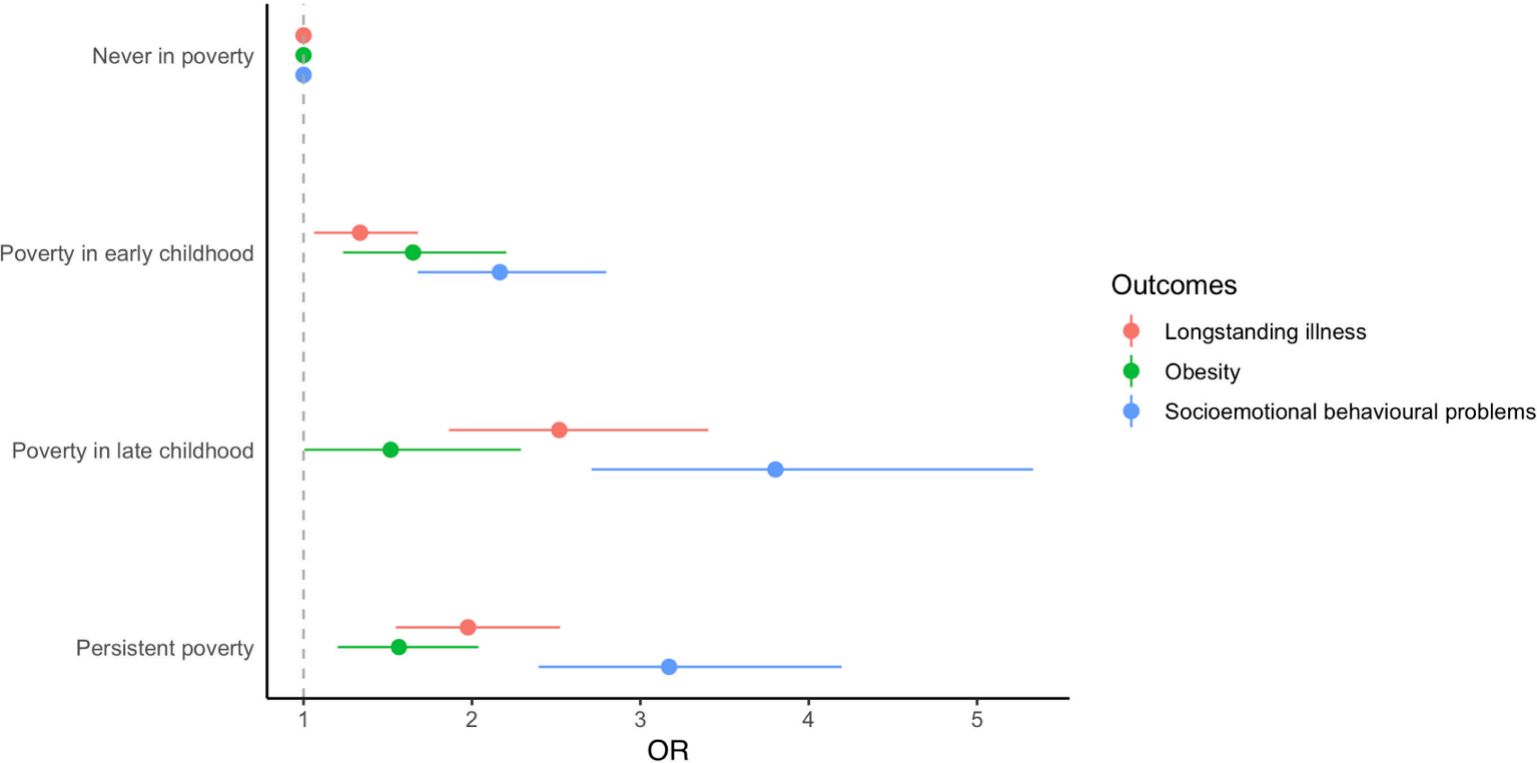
Associations of poverty trajectories and child health outcomes at age 14 years in the UK Millennium Cohort Study. Models adjusted for lone parenthood, maternal ethnicity and maternal education.

Table 2 and figure 1 shows the associations of predicted poverty trajectories and health outcomes at age 14 years. The crude analysis (model 1) showed that persistent poverty, when compared with those never in poverty, was associated with higher risk of socioemotional behavioural problems (OR: 3.97; 95% CI: 3.18 to 4.96) being obese (OR: 2.21; 95% CI: 1.80 to 2.72) and having longstanding illness (OR: 1.50; 95% CI: 1.26 to 1.79) at age 14 years. Adjustment of covariates (model 2) attenuated the effect estimates of predicted poverty trajectories and socioemotional behavioural problems and obesity but not longstanding illness, where the estimate increased (OR: 1.98; 95% CI: 1.55 to 2.52). Exposure to poverty in late childhood had a larger association with a child’s socioemotional behavioural problems and longstanding illness at age 14 years than in early childhood, but the opposite was observed for obesity. We also found that the associations of predicted poverty trajectories and child’s health outcomes did not vary by sex since all the interaction terms with sex were not statistically significant (p>0.05).

**Table 2:** Associations of predicted poverty trajectories* using latent class analysis from age 9 months to 14 years and child health outcomes at child’s age 14 years in the UK Millennium Cohort Study

OR	Model*	n	Never in poverty	Poverty in early childhood	Poverty in late childhood	Persistent poverty
Socioemotional behavioural problems (SDQ≥17)	1	10 329	Ref.	2.73 (2.16 to 3.46)	4.73 (3.44 to 6.52)	3.97 (3.18 to 4.96)
	2	9948	Ref.	2.17 (1.68 to 2.80)	3.80 (2.71 to 5.33)	3.17 (2.40 to 4.19)
Obesity	1	10 018	Ref.	2.04 (1.50 to 2.76)	1.96 (1.34 to 2.86)	2.21 (1.80 to 2.72)
	2	9640	Ref.	1.65 (1.24 to 2.20)	1.52 (1.01 to 2.29)	1.57 (1.20 to 2.04)
Longstanding illness	1	10 342	Ref.	1.20 (0.98 to 1.48)	2.01 (1.52 to 2.67)	1.50 (1.26 to 1.79)
	2	9965	Ref.	1.34 (1.06 to 1.68)	2.52 (1.86 to 3.40)	1.98 (1.55 to 2.52)

Predicted poverty trajectories:

Never in poverty: consistently not in poverty from age 9 months to age 14 years.

Poverty in early childhood: in poverty from age 9 months to age 7 years.

Poverty in late childhood: in poverty from age 11 years to age 14.

Persistent poverty: consistently in poverty from age 9 months to age 14 years.

*Model 1 is the crude model. Model 2 is model 1 additionally adjusted for lone parenthood, maternal education and maternal ethnicity.

SDQ, Strengths and Difficulties Questionnaire.

### Cumulative poverty and health in childhood: assessing dose-response

**Figure 2:**
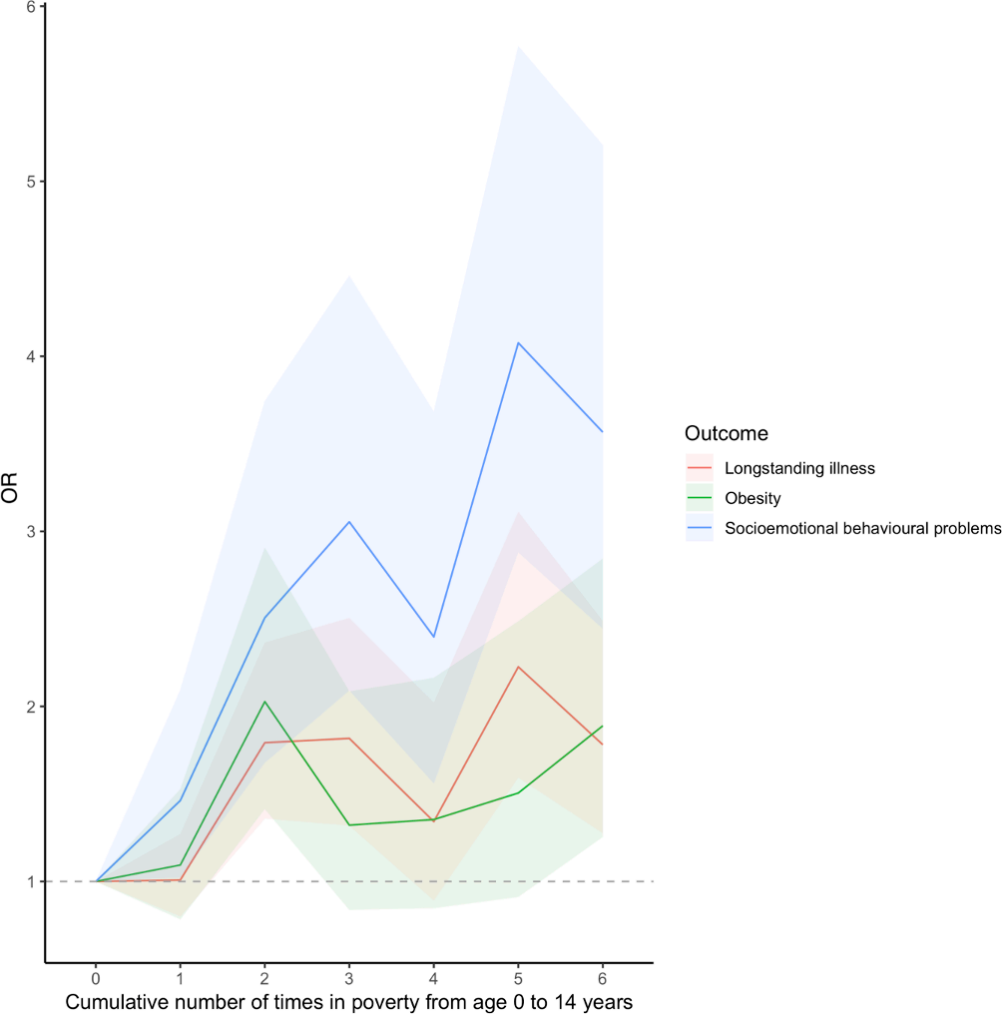
Associations of cumulative number of times in poverty from age 9 months to 14 years and child health outcomes at age 14 years in the UK Millennium Cohort Study. Models adjusted for lone parenthood, maternal ethnicity and maternal education, shaded region show 95% CIs.

We assessed whether the associations of predicted poverty trajectories and socioemotional behavioural problems, obesity and longstanding illnesses at age 14 showed any dose-response relationship using cumulative experiences of poverty. Crude models showed a more prominent dose-response relationship for socioemotional behavioural problems at age 14 (online supplementary table 3). After adjusting for covariates, the dose-response relationship for socioemotional behavioural problems at age 14 remained but attenuated (table 3, figure 2).

**Table 3:** Associations of cumulative poverty and child health outcomes at child’s age 14 years in the UK Millennium Cohort Study

Outcomes	Cumulative number of times of poverty						
	0	1	2	3	4	5	6
Socioemotional behavioural problems (SDQ≥17)	Ref.	1.46 (1.02 to 2.09)	2.51 (1.68 to 3.74)	3.05 (2.09 to 4.46)	2.40 (1.56 to 3.68)	4.08 (2.88 to 5.77)	3.57 (2.44 to 5.21)
Obesity	Ref.	1.09 (0.78 to 1.53)	2.03 (1.41 to 2.91)	1.32 (0.84 to 2.09)	1.35 (0.85 to 2.16)	1.51 (0.91 to 2.49)	1.89 (1.25 to 2.84)
Longstanding illness	Ref.	1.01 (0.80 to 1.27)	1.79 (1.36 to 2.36)	1.82 (1.32 to 2.51)	1.34 (0.89 to 2.02)	2.23 (1.59 to 3.11)	1.78 (1.27 to 2.49)

Models adjusted for lone parenthood, maternal education and maternal ethnicity.

SDQ, Strengths and Difficulties Questionnaire.

### Sensitivity analyses

The variance inflation factor (VIF) of all covariates in all models did not exceed 5. Analysis using only those with complete and using multiple imputed datasets (online supplementary tables 3 and 4) showed similar patterns with the main analysis. Sensitivity analysis using subjective poverty also showed similar patterns of associations (online supplementary table 5) as our main analysis.

## Discussion

Using a nationally representative cohort of UK children, this study showed that one in five UK children were in persistent poverty up to age 14. Any exposure to poverty was associated with worse physical and mental health in adolescence. Exposure to poverty early in childhood was associated with an increased obesity risk in adolescence, while mental health problems were more strongly associated with current experience of poverty in late childhood. Moreover, there was a clear dose-response relationship between poverty exposure and risk of mental health problems at age 14. Our findings are consistent with those from a Canadian cohort, which showed latent effects of poverty on obesity in late childhood.^11^ This study characterises children’s longitudinal experience of poverty in the UK,^1^ as well as identifying sensitive periods of poverty that may have different influence on adolescent physical and mental health.

### Strengths and limitations

This is the first study describing longitudinal patterns of poverty in the UK using a large contemporary nationally representative cohort, rich in data on family characteristics. This study, however, has several limitations. First, latent class analysis is a data-driven process, therefore predicted poverty trajectories generated by our models might not be able to be generalised to non-UK contexts. Furthermore, latent class analysis does not always generate classes that make sense from a policy perspective. For example, in our analysis while the persistent poverty class is clearly evident, there was some overlap between our early and late poverty classes, and those who were classified as ‘never in poverty’ might have experienced poverty once despite a very low probability (online supplementary figure 2 and table 6). The latent classes should therefore be interpreted with caution. Nevertheless, our predicted poverty trajectories are similar to those found in the Canadian cohort,^10 11^ suggesting that our results may be relevant to other economically developed settings. Second, as in other longitudinal studies, the issue of missing data is inevitable. We have repeated our analysis using complete cases and observed similar patterns of associations as in the main analysis, suggesting that missing data is less likely to bias our results. Third, we assessed associations between poverty trajectories and a limited number of important indicators of adolescent health, which we chose because they are important from a public health perspective, and predictive of later adult health status. Fourth, we used a measure of parent-reported longstanding illness rather than limiting longstanding illness. However, limiting longstanding illness may have a larger impact on the life of the child and the family. Lastly, we noted that a number of our measures were based on parents’ self-report. Socioemotional behavioural problems of adolescents were reported by their parents using the SDQ, which is subject to measurement error. For instance, mothers who were not in employment might experience distress and rate their child’s mental health more negatively.^18^ However, previous studies have found good inter-rater agreement between parent and teacher versions of the SDQ.^36^ Also, self-reported income might be prone to measurement error. However, previous review showed that both response bias and random error for self-reported income are quite low.^37^

### Policy implications

Our findings are important in the context of rising child health inequalities in the UK.^38^ For many children in the UK, there are concerning indications that the social conditions in which they live have deteriorated in recent years. The number of children who are living in poverty has increased, with the UN rapporteur on extreme poverty describing the situation in the UK as ‘not just a disgrace, but a social calamity and an economic disaster, all rolled into one’.^39^ At the same time, the resources available to health and social care services have reduced, limiting their capacity to respond to these adverse trends.^6^

Poor mental health in children and adolescents in the UK is of particular public health concern,^40 41^ with around 1 in 10 experiencing a diagnosable mental health disorder.^42^ Following the rising trend of child poverty, our analysis indicates that the ongoing rise in poverty is an important risk factor for poor mental health in children.^43^ As such, the impact of poverty on children’s mental health is likely to have profound implications for social policies and their associated social costs, given mental health tracks from early life to adulthood.^44^ Adolescence is the period of greatest and most rapid development after infancy.^45^ About 75% of life-time mental health disorders have their onset before age 25 years with the peak age of onset for many during adolescence, highlighting the policy importance of this period.^46^

While it is important to focus attention on the mental health and physical health needs of children and their families, Cooper and Stewart highlight that any strategy with specific targets to improve life chances for children (eg, child mental health) *without focusing on child poverty* will struggle as there will be an increase need on services as poverty levels rise.^6^ In addition, they highlighted that interventions focusing on narrower domains of specific health outcomes, while important, have relatively limited benefits, whereas increasing household income, which is connected with multiple health outcomes across the life course, is likely to have broader positive effects for mental and physical health of families.^6^

Measuring levels of poverty over time is critical in order to guide public policy, to track impacts on child health and to facilitate international comparisons. Multiple reports have outlined how child poverty can be tackled in the UK, particularly by central and local government. All suggest similar strategies (see online supplementary appendix),^38 47–52^ for example, they describe the need for a renewed commitment by UK government to prioritise ending child poverty; reporting on income child poverty data; protecting child benefits with a ‘triple lock’, as has been done with pensions for the elderly.

On a local level, health professionals are in a unique position to prevent the health consequences of child poverty but they require the tools and resources to do so effectively.^52–54^ Evidence is emerging that systematic screening for social determinants of health has a positive impact on families in poverty, and can improve health outcomes for children.^52^ Furthermore, health professionals can advocate for children’s rights to health. All children are entitled to the best possible health, as enshrined by the United Nations Convention on the Rights of the Child. The UK signed the convention, ratified it in the early 1990s.^55^ The UK has also committed to achieve the Sustainable Development Goals, a collection of 17 global goals set by the United Nations General Assembly, with a commitment to eradicate poverty and reduce inequality nationally and internationally.^56^ Health professionals are therefore well-placed to argue that policies and services in the UK should fulfil our moral and legal responsibility to ensure that every child is able to achieve their full potential.
