# Supplementary material for: Poverty dynamics and health in late childhood in the UK: evidence from the Millennium Cohort Study
Source: Arch Dis Child. 2019 Jun 11;104(11):1049–55. doi: 10.1136/archdischild-2018-316702 (PMC6837248; doi:10.1136/archdischild-2018-316702)
Supplement: Supplementary file 1 [file archdischild-2018-316702supp001.pdf]

Supplementary materials

Supplementary Figure 1: Directed acyclic graph for the current study

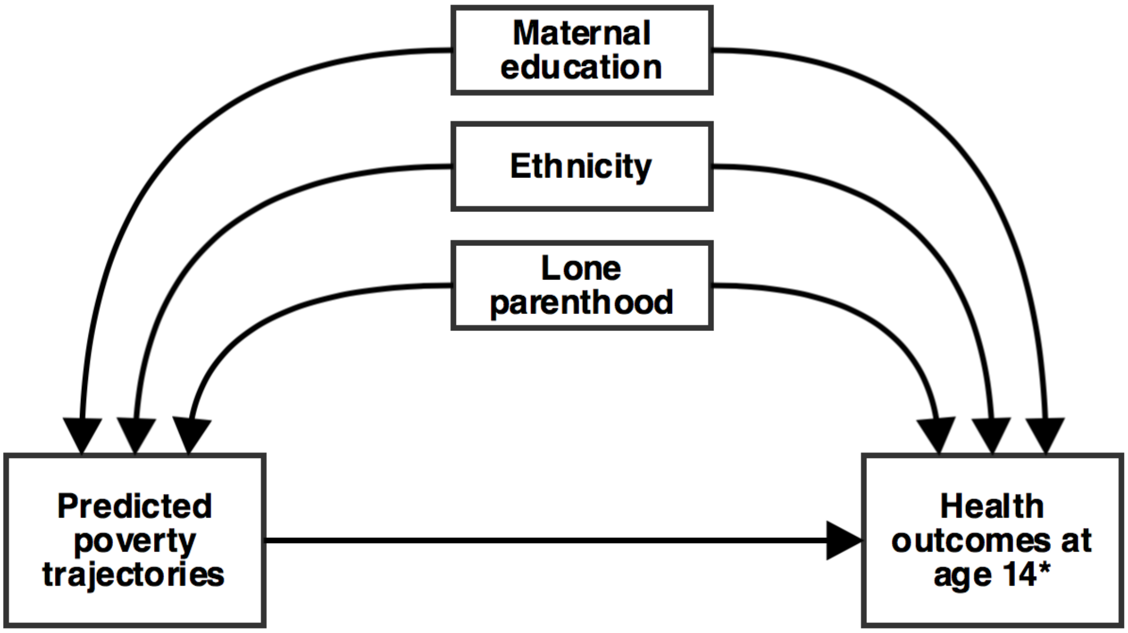

*Health outcomes included socioemotional problems, obesity and longstanding illnesses.

Supplementary Figure 2: Predicted probability for families in the MCS being in poverty at each wave of follow-up in different predicted poverty trajectories

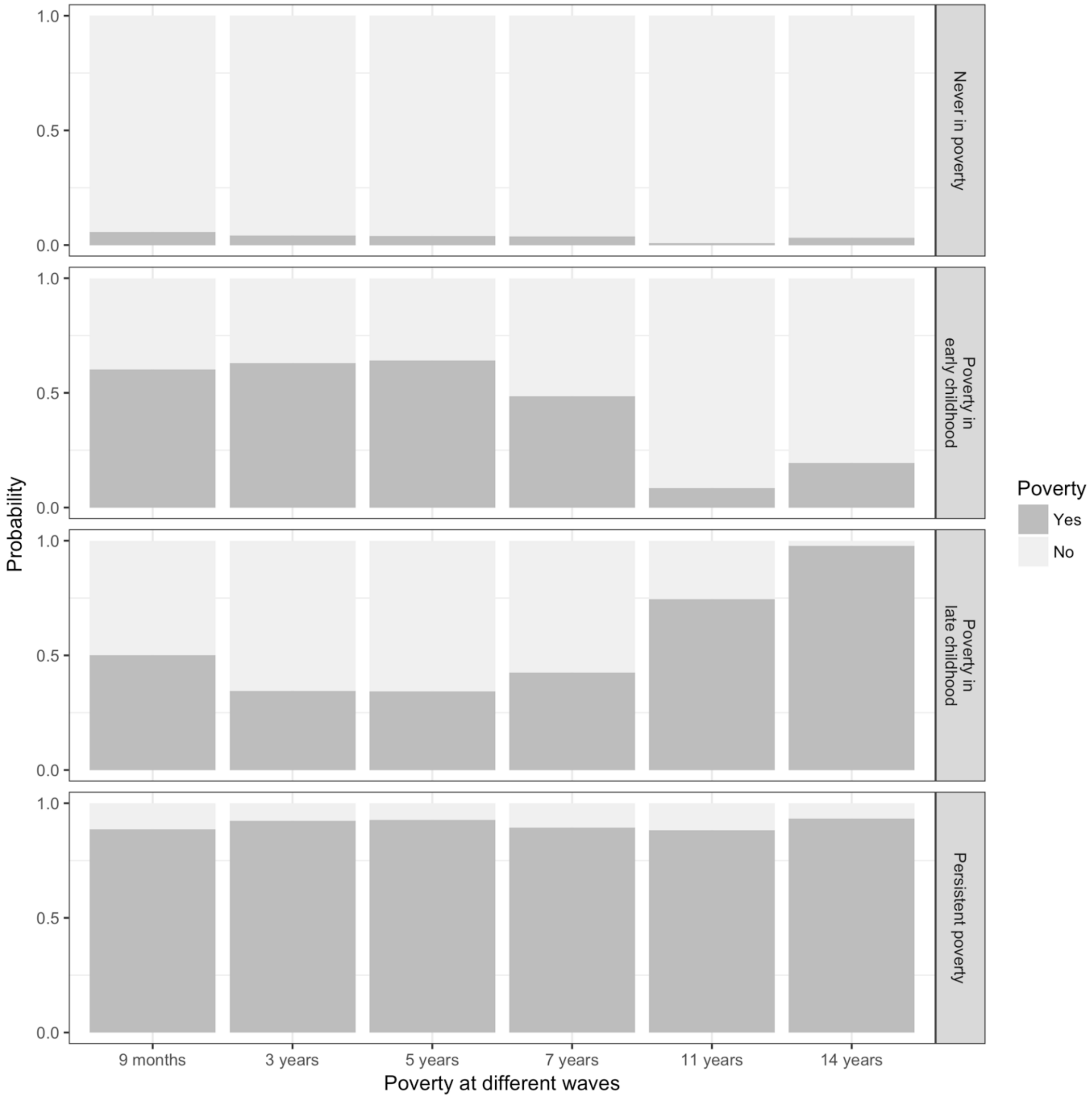

The figure shows the latent class model density estimate of the percentage of people in the underlying population in poverty at each follow-up time point in each of the predicted poverty trajectories. We took a cut-off of probability of 0.5 to consider whether families in a particular trajectory at a given time are in poverty.

Supplementary Figure 3: Flow diagram showing inclusion/exclusion of cohort participants into the analysis

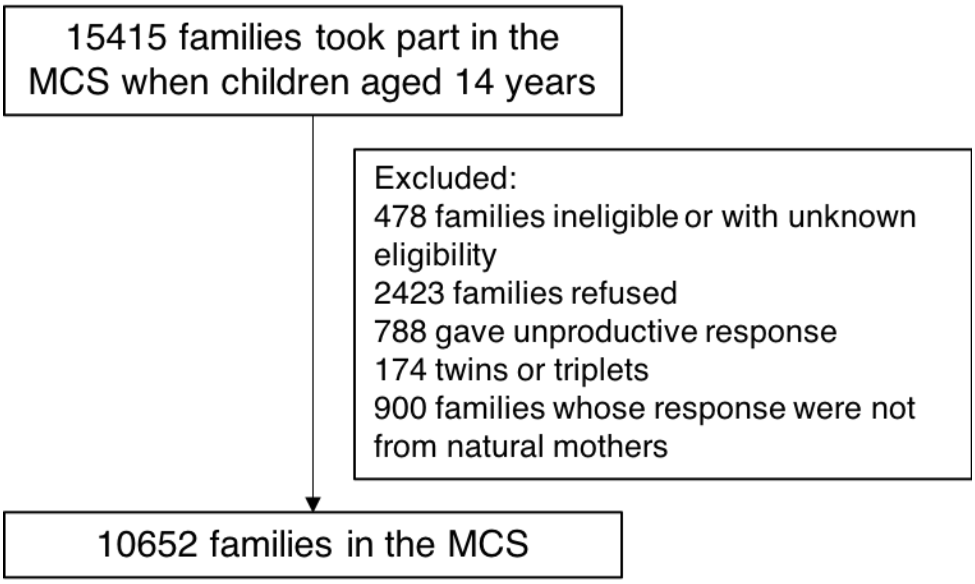

Supplementary Table 1: Comparison between cohort participants with complete and missing observations

	Participants with missing data	Participants with complete observations	Cohen's effect size
n	1506	9146	
Predicted poverty trajectories (%)			0.36
Never in poverty	738 (49.0)	5914 (64.7)	
Poverty in early childhood	224 (14.9)	1200 (13.1)	
Poverty in late childhood	94 (6.2)	436 (4.8)	
Persistent poverty	450 (29.9)	1596 (17.5)	
Socioemotional problem (SDQ≥17) (%)	291 (24.6)	711 (7.8)	0.58
Obese (%)	73 (8.4)	664 (7.3)	0.04
Longstanding illness (%)	186 (15.6)	1261 (13.8)	0.05
Maternal education (%)			0.27
Higher degree	30 (2.7)	388 (4.2)	
First degree	122 (10.8)	1490 (16.3)	
Diploma	85 (7.5)	897 (9.8)	
A-levels	88 (7.8)	947 (10.4)	
GCSE A-C	394 (34.9)	2989 (32.7)	
GCSE D-G	127 (11.3)	884 (9.7)	
None	282 (25.0)	1551 (17.0)	
Ethnicity (%)			0.01
White	954 (84.6)	7748 (84.7)	
Mixed	18 (1.6)	74 (0.8)	
Indian	22 (2.0)	241 (2.6)	
Pakistani and Bangladeshi	70 (6.2)	646 (7.1)	
Black or Black British	54 (4.8)	272 (3.0)	
Other ethnic groups	9 (0.8)	165 (1.8)	
Lone parenthood at 9 months	48 (4.2)	240 (2.6)	0.10
Male	487 (42.4)	4656 (50.9)	0.17

Supplementary table 2: Weighted number of families in poverty in each wave of follow-up

Follow-up time	Weighted count (%)
9 months	2702.6 (27.0)
3 years	2821.3 (28.0)
5 years	2845.6 (28.3)
7 years	2809.2 (28.1)
11 years	2915.4 (29.1)
14 years	2769.0 (27.6)

The following weighing variables were used in producing this table: the stratum variable is pttype2 to account for stratified design of MCS; the clustering variable is sptn00 to account for data being clustered at ward level; the finite population correction factor is nh2 to measure how much extra precision we could achieve when the sample size become close to population size; and the survey weight is fovwt2 to account for attrition.

Supplementary Table 3: Associations of cumulative poverty and child health outcomes at child's age 14 years in the UK Millennium Cohort Study

		Cumulative number of times of poverty						
**Model**	**Outcomes**	**0**	**1**	**2**	**3**	**4**	**5**	**6**
**A**	Socioemotional behavioural problems (SDQ ≥ 17)	Ref.	1.71 (1.19 to 2.44)	3.04 (2.08 to 4.46)	3.91 (2.76 to 5.54)	3.08 (2.05 to 4.62)	5.06 (3.70 to 6.91)	4.37 (3.17 to 6.03)
	Obesity	Ref.	1.27 (0.92 to 1.75)	2.48 (1.72 to 3.59)	1.66 (1.05 to 2.63)	1.75 (1.10 to 2.80)	2.03 (1.31 to 3.14)	2.61 (1.81 to 3.77)
	Longstanding illness	Ref.	0.94 (0.75 to 1.18)	1.60 (1.23 to 2.08)	1.62 (1.17 to 2.24)	1.16 (0.80 to 1.69)	1.82 (1.38 to 2.40)	1.32 (1.02 to 1.70)
**B**	Socioemotional behavioural problems (SDQ ≥ 17)	Ref.	1.48 (0.98 to 2.25)	2.34 (1.48 to 3.70)	2.75 (1.73 to 4.38)	2.02 (1.23 to 3.31)	3.77 (2.51 to 5.68)	2.70 (1.64 to 4.44)
	Obesity	Ref.	1.12 (0.79 to 1.57)	1.98 (1.36 to 2.89)	1.39 (0.85 to 2.27)	1.28 (0.76 to 2.15)	1.61 (0.99 to 2.63)	2.19 (1.41 to 3.41)
	Longstanding illness	Ref.	1.00 (0.78 to 1.28)	1.80 (1.35 to 2.40)	2.01 (1.40 to 2.88)	1.52 (1.02 to 2.26)	2.06 (1.46 to 2.91)	1.90 (1.29 to 2.79)
**C**	Socioemotional behavioural problems (SDQ ≥ 17)	Ref.	1.41 (0.92 to 2.15)	2.50 (1.70 to 3.69)	3.06 (2.07 to 4.52)	2.95 (1.91 to 4.57)	3.53 (2.19 to 5.69)	3.49 (2.33 to 5.22)
	Obesity	Ref.	1.18 (0.80 to 1.76)	1.97 (1.28 to 3.03)	1.47 (0.90 to 2.39)	1.56 (0.94 to 2.60)	1.49 (0.96 to 2.32)	1.76 (1.23 to 2.52)
	Longstanding illness	Ref.	1.03 (0.78 to 1.37)	1.70 (1.31 to 2.22)	1.78 (1.29 to 2.47)	1.47 (0.99 to 2.18)	2.33 (1.62 to 3.34)	1.94 (1.38 to 2.72)

Model A shows the crude associations of cumulative poverty and child health outcomes at child's age 14.

Model B shows complete case analysis (n=7475). The model adjusted for lone parenthood, maternal education and maternal ethnicity.

Model C shows analysis using multiple imputed datasets (n=10652). The model adjusted for lone parenthood, maternal education and maternal ethnicity.

Supplementary Table 4: Associations of predicted poverty trajectories using latent class analysis from age 9 months to 14 years and child health outcomes at child's age 14 years in the UK Millennium Cohort Study

**Models**	**Odds ratio (OR)**	**Never in poverty**	**Poverty in early childhood**	**Poverty in late childhood**	**Persistent poverty**
A	Socioemotional behavioural problems (SDQ ≥ 17)	Ref.	2.10 (1.52 to 2.89)	2.82 (1.87 to 4.25)	3.03 (2.16 to 4.27)
	Obesity	Ref.	1.62 (1.19 to 2.21)	1.61 (1.02 to 2.53)	1.72 (1.23 to 2.41)
	Longstanding illness	Ref.	1.35 (1.03 to 1.77)	2.99 (2.11 to 4.22)	1.91 (1.42 to 2.57)
B	Socioemotional behavioural problems (SDQ ≥ 17)	Ref.	2.17 (1.68 to 2.80)	3.74 (2.68 to 5.22)	3.12 (2.36 to 4.13)
	Obesity	Ref.	1.65 (1.23 to 2.23)	1.51 (0.99 to 2.31)	1.54 (1.15 to 2.06)
	Longstanding illness	Ref.	1.38 (1.10 to 1.75)	2.60 (1.95 to 3.47)	2.09 (1.63 to 2.68)

Models adjusted for lone parenthood, maternal education and maternal ethnicity. Model A is complete cases analysis (n=9146). Model B is analysis using multiple imputed datasets (n=10652).

Supplementary Table 5: Associations of predicted *subjective* poverty trajectories using latent class analysis from age 9 months to 14 years and child health outcomes at child's age 14 years in the UK Millennium Cohort Study

**Odds ratio (OR)**	**Never in poverty**	**Poverty in early childhood**	**Poverty in late childhood**	**Persistent poverty**
Socioemotional behavioural problems (SDQ ≥ 17)	Ref.	2.20 (1.65 to 2.92)	2.52 (1.89 to 3.35)	3.51 (2.79 to 4.42)
Obesity	Ref.	1.68 (1.23 to 2.29)	1.56 (1.16 to 2.09)	2.06 (1.58 to 2.70)
Longstanding illness	Ref.	1.39 (1.10 to 1.76)	1.56 (1.27 to 1.93)	1.67 (1.39 to 2.01)

Models adjusted for lone parenthood, maternal education and maternal ethnicity.

Supplementary table 6: Weighted number of times of poverty of the cohort participants of the UK Millennium Cohort Study

Number of times in poverty	Weighted count (%)
0	3802.9 (49.5)
1	1015.3 (13.2)
2	560.9 (7.3)
3	512.1 (6.7)
4	505.9 (6.6)
5	517.5 (6.7)
6	764.6 (10.0)

Supplementary text box: Key recommendations to address child poverty in recent reports[1-7]

Central and local government:

- Recommit to ending child poverty by making it a national priority[1]
- Recommit to record and report income child poverty[2]
- Adopt a "child health in all policies" approach to decision making and policy development[2]
- Ensure universal early years public health services are prioritised and supported financially, with targeted help for children and families in poverty[2]
- Review and implement systems of taxation, benefits, pensions and tax credits that provide a minimum living income for healthy lives, and reinstate the link between annual increases in benefit levels and inflation[3-6]
- Protect families from rising living costs by restoring the value of children's benefits and protecting them with a triple lock, and providing free school meals for children in low income households[1]
- End costly delays and poor decision-making in the benefits system[1]
- Reform childcare/early years education to improve the quality, affordability and availability of childcare[1,5]
- Grant local government a greater role in deciding how public resources are used to improve the health and well-being of the communities they serve[4]
- Establish a commission to consider how businesses can ensure that their customers on a low income do not face paying the highest prices for goods and services[5,6]

Public Health England, Clinical Commissioning Groups and other National Health Service (NHS) agencies

- NHS Digital need to develop standards to ensure child health data are of high quality, captured to pre-specified definitions, and analysed consistently across the UK[2]
- Provide leadership to support health services and clinicians to reduce children's exposure to poverty and its consequences[4]
- Encourage the provision of services in primary care to reduce poverty among people with chronic illness[4]

- Introduce systematic screening and referring for social determinants of health within primary care[7]

References

1. Child Poverty Action Group. A programme for government 2015-2020. London: Child Poverty Action Group, 2015.
2. Royal College of Paediatrics and Child Health. State of Child Health Report 2017. In: Viner RM, ed. London: Royal College of Paediatrics and Child Health, 2017.
3. Marmot M, Atkinson T, Bell J, et al. Fair Society Healthy Lives: The Marmot Review. London: UCL Institute of Health Equity, 2010.
4. Whitehead M, Bambra C, Barr B, et al. Due North: Report of the Inquiry on Health Equity for the North. Liverpool, U.K.: Centre for Local Economic Strategies, University of Liverpool, 2014.
5. Joseph Rowntree Foundation. We can solve poverty in the UK. York: Joseph Rowntree Foundation, 2016.
6. End Child Poverty Coalition. Feeling the Pinch. London: End Child Poverty Coalition, 2017.
7. Chung EK, Siegel BS, Garg A, et al. Screening for Social Determinants of Health Among Children and Families Living in Poverty: A Guide for Clinicians. *Current problems in pediatric and adolescent health care* 2016;46(5):135-53. doi: 10.1016/j.cppeds.2016.02.004 [published Online First: 2016/04/23]
